# World Health Organization Methodology to Prioritize Emerging Infectious Diseases in Need of Research and Development

**DOI:** 10.3201/eid2409.171427

**Published:** 2018-09

**Authors:** Massinissa Si Mehand, Piers Millett, Farah Al-Shorbaji, Cathy Roth, Marie Paule Kieny, Bernadette Murgue

**Affiliations:** World Health Organization, Geneva, Switzerland (M. Si Mehand, P. Millett, F. Al-Shorbaji, M.P. Kieny, B. Murgue);; Department for International Development, London, UK (C. Roth)

**Keywords:** severe emerging infectious diseases, prioritization, multicriteria decision analysis, expert opinion, multidisciplinary method, World Health Organization, R&D Blueprint, epidemics, Ebola virus, Marburg virus infection, Middle East respiratory syndrome coronavirus, severe acute respiratory syndrome, Lassa virus, Nipah virus, Rift Valley fever, Zika virus, Crimean-Congo hemorrhagic fever, severe fever with thrombocytopenia syndrome, South American hemorrhagic fever, plague, hantavirus, viruses, bacteria

## Abstract

The World Health Organization R&D Blueprint aims to accelerate the availability of medical technologies during epidemics by focusing on a list of prioritized emerging diseases for which medical countermeasures are insufficient or nonexistent. The prioritization process has 3 components: a Delphi process to narrow down a list of potential priority diseases, a multicriteria decision analysis to rank the short list of diseases, and a final Delphi round to arrive at a final list of 10 diseases. A group of international experts applied this process in January 2017, resulting in a list of 10 priority diseases. The robustness of the list was tested by performing a sensitivity analysis. The new process corrected major shortcomings in the pre–R&D Blueprint approach to disease prioritization and increased confidence in the results.

Recent outbreaks of Ebola virus disease, Middle East respiratory syndrome, and Zika virus disease illustrate that emerging infectious diseases will continue to cause major public health emergencies. Further work is needed to strengthen defenses with medical countermeasures (MCMs) and other protective interventions. Building on recent experiences and at the request of the World Health Assembly in May 2015 (*1*), the World Health Organization (WHO) launched the R&D Blueprint for action to prevent epidemics. This global strategy and preparedness plan is designed to ensure that targeted research and development (R&D) will strengthen emergency response by accelerating availability of biomedical technologies to populations and patients during epidemics (*2*). The R&D Blueprint focuses on severe emerging diseases that pose a major risk for causing a public health emergency and for which MCMs or substantial R&D initiatives and pipelines are insufficient or nonexistent (*3*).

Experts compiled an initial list of relevant diseases at an informal consultation in December 2015 (*4*). A more robust methodology was needed, one that could be standardized and repeated regularly for reviewing and, if necessary, updating the list in the light of successful development of new interventions or the emergence of new disease threats.

WHO settled on a 3-pronged approach: 1) a methodology development and review process; 2) an annual review of a list of prioritized diseases; and 3) a decision instrument to guide decision-making on a novel disease (Technical Appendix 1). All 3 processes use a common set of weighted criteria and subcriteria, such as the human-to-human transmissibility of the disease or its potential societal impact (Technical Appendix 2). This process is inherently expert-driven because the R&D Blueprint addresses pathogens that are yet to be fully characterized and for which an understanding of how to diagnose, prevent, and treat the resulting diseases is incomplete. Further, these pathogens might behave differently on different occasions because of variation in the biologic, cultural, or environmental context. Decisions have to be made on the basis of partial information supplemented by expert opinion. Any methodology will be prone to biases (*3*).

This article assesses the application of this methodology for the 2017 annual review of the WHO R&D Blueprint priority list of diseases. We consider its effectiveness and assess the degree of confidence that can be placed in the list produced.

## Developing a Prioritization Process

WHO developed a comprehensive methodology (*3*) to ensure the list of the R&D Blueprint prioritized diseases best reflects targeted global health needs and focuses on the most pressing threats. The approach taken drew heavily on established best practice (*5*–*7*) and is based on practical national and regional experiences in compiling similar lists (*8*–*14*). This approach also specifically addressed criticism of pre–R&D Blueprint attempts by WHO to prioritize diseases by developing tools for assessing confidence in the results generated and addressing potential biases (*5*).

Disease prioritization is not a straightforward task and requires a defined set of criteria on which to base prioritization (*7*). These criteria can be qualitative, intangible, or subjective, changing for different stakeholders (*15*). The criteria can also be interdependent, complicating separate assessment (*16*). For instance, the case-fatality rate of a disease has a social effect, which in turn has an economic effect. Given the complexity and the challenges of disease prioritization, ensuring the process is transparent and reproducible is important (*5*,*7*,*17*).

Recent disease prioritization methods were summarized in a 2015 review by the European Centre for Disease Prevention and Control (ECDC) (*5*), which extrapolated a series of best practices. Several subsequent studies were also identified (Technical Appendix 3). Past disease prioritization studies have been conducted for different purposes, such as communicable diseases surveillance (*18*,*19*), biosecurity (*20*), and resource allocation (*21*,*22*), and have covered disease in humans, livestock, or wildlife. Many studies were conducted primarily at national (*8*–*10*,*19*,*21*–*36*) and regional (*11*–*14*,*16*,*20*,*37*–*43*) levels (e.g., Europe and North America) but rarely at a global level (*44*,*45*). None of the disease prioritization exercises matched the aims of the R&D Blueprint, its public health focus, and its global reach; thus, WHO needed to develop its own methodology.

Several different disease prioritization methods exist (*5*), including Delphi processes (*38*,*40*), multicriteria decision analysis (MCDA) (*14*,*26*,*28*,*36*,*46*), H-index (*42*,*43*), questionnaires (*11*,*13*,*22*), and qualitative algorithms (*47*,*48*). Each method has its own strengths, weaknesses, and context-dependent utility, but 3 methods most closely matched the requirements of the R&D Blueprint (*5*): 1) a semiquantitative Delphi process to narrow the list of diseases under consideration; 2) MCDA to rank the remaining diseases (Technical Appendix 4); and 3) questionnaires in the form of online survey tools to standardize information gathering from participating experts.

## Methods and Tools

The resulting methodology was developed over a year-long process, involving informal consultations, internal and external expertise, and guidance from the R&D Blueprint Scientific Advisory Group (*4*). Methods and tools were subsequently reviewed and validated by an external group of experts (*49*) and used in the review of the list of priority diseases in January 2017 (*50*).

### Prioritization Committee

Selecting the right group of experts is critical for ensuring an outcome as accurate as possible (*39*,*51*). Gathering a diverse field of expertise with a broad geographic distribution, including an in-depth knowledge of the diseases and pathogens being considered, is important. The multidisciplinary committee convened for the 2017 annual review included 24 experts drawn from Africa, Asia, Europe, North America, and South America (Technical Appendix 2). The persons present at the meeting covered all 7 areas of expertise detailed in the methodology (Technical Appendix 1) (*3*). To ensure the process was as transparent as possible, representatives from several additional organizations were present, including the World Organisation for Animal Health (OIE), which helped ensure a One Health approach was followed, as well as the Coalition for Epidemic and Preparedness Innovations and the Global Research Collaboration for Infectious Disease Preparedness, which facilitated cooperation, coordination, and the sharing of experiences outside of WHO. To minimize bias related to expert opinions, the prioritization committee is changed yearly (*7*).

### Triage of the Diseases

To narrow the list of potential priority diseases, a 2-step semiquantitative Delphi technique was adapted from established environmental horizon scanning methods (*52*). Each proposed disease was scored from 0 to 1,000, where 1,000 represented a perfect fit for the R&D Blueprint and 0 represented diseases with no epidemic potential, diseases for which effective and commercially available MCMs exist, or both. 

### Disease Scoring

**Figure 1:**
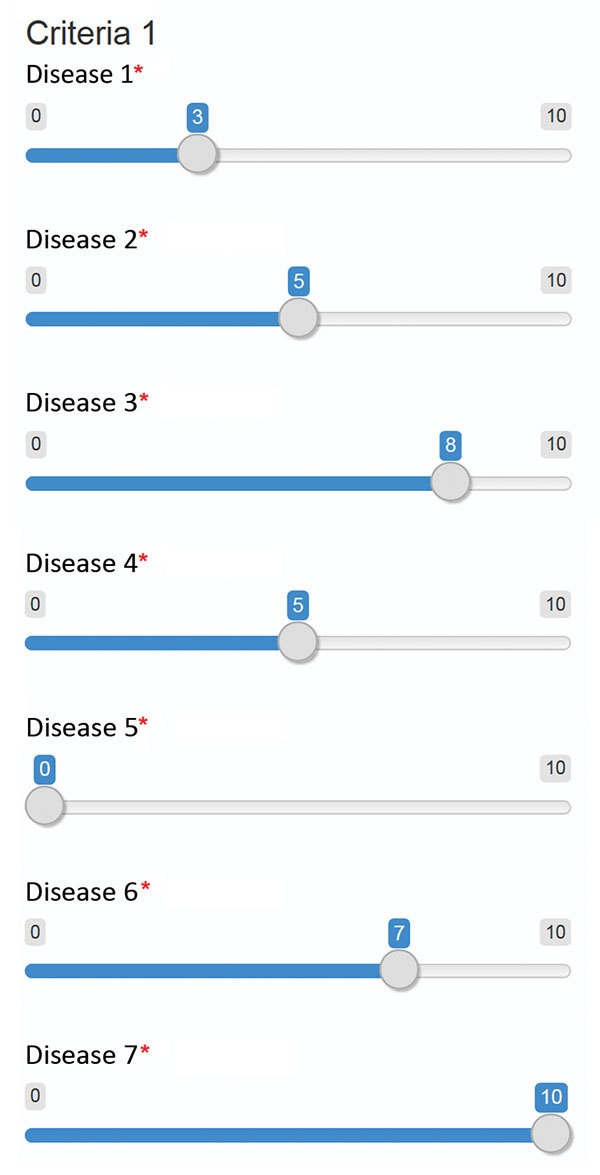
Example screenshot of tool developed in R Shiny (https://shiny.rstudio.com) using the slide bar function to compare candidate diseases for each criteria and subcriteria considered in the development of the World Health Organization R&D Blueprint to prioritize emerging infectious diseases in need of research and development. Experts were requested to compare candidate diseases to each other for each criteria, placing them in ranked order according to their knowledge. The World Health Organization Secretariat explained the meaning of the scale (0–10) to the experts before the survey. Asterisks indicate that an answer is required for each disease.

To rank the short list of diseases, an online survey tool was designed by using the slide-bar function of R Shiny (https://shiny.rstudio.com) (Figure 1). Because absolute scoring scales require broadly accepted standards (*53*) and these standards are not evident in the context of emerging infectious disease, for which many characteristic remain unclear or unknown, the WHO tool makes use of a relative scale that compares values between diseases rather than against absolute values (i.e., the impact of scoring diseases A and B at 3 and 5 is the same as scoring the same diseases at 7 and 9).

The data collected were processed by an in-house program implemented in R Studio*.* A custom analytic hierarchy process (AHP) implementation was used to calculate disease scores for each subcriterion (Technical Appendix 5). This process included normalization and weighting procedures. Comparison matrices were built from data provided by each expert and then averaged by using the geometric average (*54*). Disease scores for each subcriterion were computed, and an overall multicriteria score for each disease was ultimately computed by using the disease scores and criteria weights. Following best practices, the criteria definition and weighting steps were separated from the disease scoring (*5*,*7*,*14*,*36*,*38*). The criteria were defined in 2015 by a group of experts (*4*) and were then reviewed, validated, and weighted by another group (*49*).

### Sensitivity Analysis and Confidence Estimation

Past prioritization processes have used sensitivity analysis, commonly with lower and upper 95% CIs (*31*). Other processes included modifying the weight of criteria used (*9*,*55*) and removing them one at a time (*55*). We describe a series of sensitivity analyses, including setting all the criteria at the same weight, removing 1 criterion at a time, increasing the weight of each criterion by 20%, and doubling the weight of a criterion. This approach to sensitivity analysis enables assessment of the impact of different scenarios on the final disease ranking and provides important insights into the robustness of the ranking and the impact of potential biases (*9*,*55*).

As a confidence indicator, differences among expert opinions were considered. The arithmetic average scores and the corresponding SDs for each disease were calculated and tracked through the process by using an error propagation technique (Technical Appendix 5).

## Results

### Compiling and Reviewing a Long List of Diseases

The long list of diseases was drawn from diseases identified as requiring urgent R&D support in the 2015 priority list, diseases recommended but not included by the 2015 consultation, and diseases suggested by participants in the 2017 review. As a result, 8 diseases on the original 2015 list were supplemented by another 10 diseases. In 2017, no additional disease was selected by the decision instrument.

**Figure 2:**
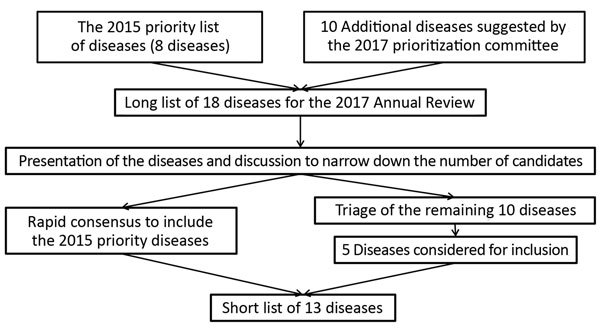
Process for compiling the short list of diseases for inclusion in the World Health Organization R&D Blueprint to prioritize emerging infectious diseases in need of research and development.

Each of these 18 diseases was then considered in turn. Two experts introduced each of the diseases on the 2015 list and those selected at the 2015 consultation. A single expert introduced each of the diseases proposed by the 2017 committee. Consensus was rapidly reached that diseases on the 2015 list should be reassessed by using the MCDA tool. A triage of the remaining 10 diseases was then carried out. The results were discussed in detail, and a further 5 diseases were added to the short list (Figure 2). Additional considerations of those diseases not incorporated into the list were also discussed (Technical Appendix 6), such as the importance of continuing relevant R&D (*50*).

### Ranking the Short List of Diseases

**Figure 3:**
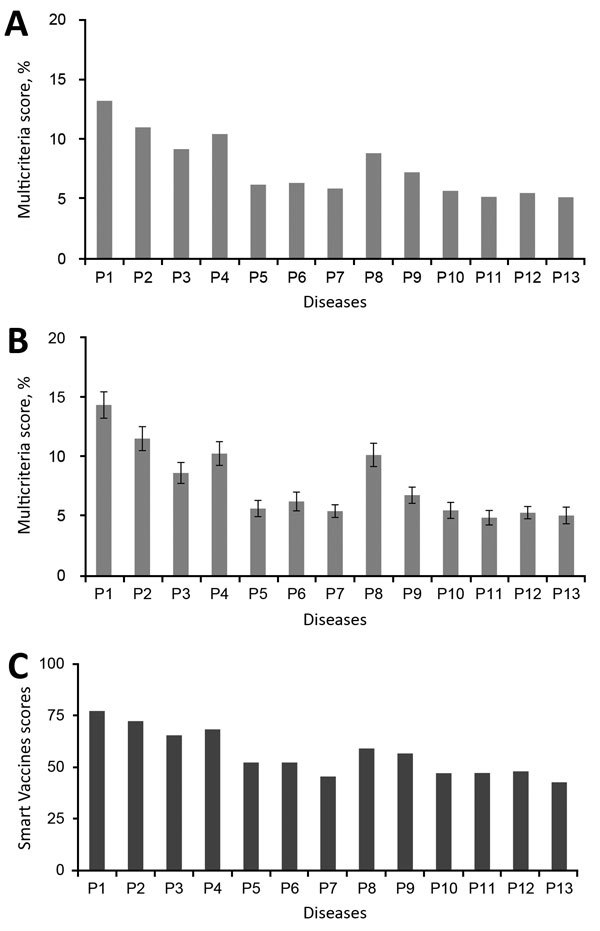
Multicriteria scores of diseases considered in the 2017 prioritization exercise for the development of the World Health Organization R&D Blueprint to prioritize emerging infectious diseases in need of research and development. A) Disease final ranking using the geometric average of the comparison matrices. B) Disease final ranking using the arithmetic average of the raw data. Error bars correspond to SD, indicating disagreement among experts. C) Disease final ranking using the SMART Vaccines prioritization tool (*56*). P1, Ebola virus infection; P2, Marburg virus infection; P3, Middle East Respiratory Syndrome coronavirus infection; P4, severe acute respiratory syndrome; P5, Lassa virus infection; P6, Nipah virus infection; P7, Rift Valley fever; P8, Zika virus infection; P9, Crimean-Congo hemorrhagic fever; P10, severe fever with thrombocytopenia syndrome; P11, South American hemorrhagic fever; P12, plague; P13, hantavirus infection.

The MCDA tool was used to generate 1) scores for each disease against each subcriterion; 2) aggregated scores for each criterion for each disease; and 3) multicriteria scores for each disease. The aggregated scores for each criterion for each disease were considered in more depth (*50*). The multicriteria scores (Figure 3, panel A) were then used to rank diseases on the short list. Six diseases (P1, P2, P3, P4, P8, and P9) were highly ranked; a group of 3 diseases (P5, P6, and P7) were ranked next, and the final 4 diseases (P10, P11, P12, and P13) had the lowest ranking.

A consensus was quickly reached that the group of 6 top-ranking diseases (P1, P2, P3, P4, P8, and P9) should be on the 2017 priority list. An uncertainty analysis revealed overlapping results for the remaining pathogens, particularly noticeable for the lower 2 tiers (Figure 3, panel B). As a result, the multicriteria scores alone were insufficient to differentiate between the remaining 8 diseases. An additional round of the Delphi technique enabled the committee to compile a final list: arenaviral hemorrhagic fevers (including Lassa fever); Crimean-Congo hemorrhagic fever; filoviral diseases (including Ebola and Marburg virus infections); Middle East respiratory syndrome coronavirus infection; Nipah virus infection and related henipaviral diseases; other highly diseases coronaviral diseases (such as severe acute respiratory syndrome); Rift Valley fever; severe fever with thrombocytopenia syndrome; and Zika virus infection. A more detailed discussion as to how the list was compiled can be found in the WHO report on the 2017 prioritization exercise (*50*).

### Assessing Confidence in the Results

The multiscenario sensitivity analysis detailed the influence of each criterion on the final ranking. When all the criteria were set at the same weight as those used in a similar exercise conducted in Kenya (*9*), the multicriteria scores were affected, but the overall ranking remained largely the same, with 2 diseases (P5 and P7) switching positions.

When highly weighted criteria, such as human-to-human transmissibility, were suppressed, major changes in the multicriteria scores were observed, but a much smaller impact was evident on the overall disease ranking. A notable exception was when the MCMs criterion was suppressed, after which no notable impact on the multicriteria scores or the final ranking was observed.

When the weight of each criterion was increased by 20%, no notable changes in ranking were observed. Doubling the weights of highly weighted criteria resulted in changes in the overall multi criteria scores but had a minimal impact on the overall ranking of diseases. Once again, doubling the weight of the MCMs criterion had minimal impact on the multicriteria scores and the overall disease ranking.

To further validate the 2017 priority list, the same data were analyzed by using the SMART Vaccines prioritization tool (*56*). Unlike the methodology discussed in this article, the SMART Vaccines tool makes use of absolute rather than relative values. This feature precludes a direct comparison of specific results but helps explore the reproducibility of the list as a whole. The results from the SMART Vaccines prioritization tool also grouped the same diseases together in the same 3 tiers (Figure 3, panel C).

## Discussion

The 2017 annual review resulted in a list of diseases that pose a risk for a public health emergency and for which an urgent need for R&D exists (*50*). The earlier ECDC review highlighted numerous general weaknesses among published prioritization processes (*5*). It identified shortcomings in WHO approaches toward disease prioritization before the R&D Blueprint, including insufficient detail in reporting; a lack of transparency, in particular as to how the prioritization criteria were developed; a need for greater consideration on sources of bias; a better discussion of implementation challenges; methodologic anomalies, such as the use of only a single round of the Delphi technique; and a lack of external review of the methodology and subsequent publications.

The methodology developed by WHO for the R&D Blueprint explicitly addresses these shortcomings (e.g., mitigation of numerous sources of bias). Past methodologic anomalies have also been addressed (e.g., a 2-step semiquantitative Delphi technique is now being used). The reporting process has been strengthened; the methodology has been published in full (*3*), as has a detailed report of its use during the 2017 annual review (*50*).

The new approach is also much more transparent, with all publications being more detailed and openly available. These publications explain the reasoning behind why certain diseases ultimately were (or were not) included on the list. The process by which the prioritization criteria were developed (Technical Appendix 2) is also well documented in meeting reports (*4*,*49*).

Shortcomings in the review process have been addressed, in part, by separate committees to develop and implement the methodology because these committees effectively review each other’s work. The methodology itself was validated through a dedicated expert consultation, improving the review procedures further. Finally, publication of this article further expands opportunities to review the approach and its implementation.

Several challenges to implementation exist. Other prioritization studies have invested extensive resources into identifying potentially relevant diseases. For example, Cox et al. conducted a bibliometric analysis of >3,000 infectious organisms in North America to identify the 651 pathogens relevant to their study (*43*). In Belgium, Cardoen et al. complimented a literature review with expert consultations (*36*). In the Netherlands, Havelaar et al. went a step further, supplementing their literature review with consultations with international, regional, and national experts to identify the relevant subset of pathogens (*26*).

At present, the community proposing additional diseases to be considered in an annual review of the R&D Blueprint is limited. To address this issue in future prioritization exercises and to better reflect regional factors in the long list of diseases, the WHO regional offices will be more actively involved in the inclusion of a wider range of experts.

The next methodology review should look at the reproducibility of these tools. In the interim, further improvement of the MCDA model might include reviewing the pertinence of the MCMs criterion given that it had little effect on the multicriteria scores of the diseases, reweighting the criteria, drawing on a wider community of relevant expertise and a larger sample size, and reviewing and simplifying the specific wording of the subcriteria.

The R&D Blueprint methodology was developed to mitigate numerous sources of bias, including flaws in study design, selection bias, interviewer bias, chronology bias, and recall bias (*3*). These efforts were largely successful; however, further work might be necessary to mitigate selection and recall bias.

The selection of experts to participate in the MCDA is important for mitigating selection biases. WHO’s policies on geographic and gender representation go some way to address selection bias. Considerable resources were also expended to create a committee with the diverse range of expertise required, with experts from microbiology and virology, clinical management of severe infections, epidemiology and outbreak investigation and response, public health policy, animal health, mathematical modeling of disease, environmental and social science, nongovernmental organizations, and the security sector. This diversity is consistent with and exceeds the range of participants found in other studies, allowing for some variation based on their specific purposes (*8*,*9*,*11*,*13*,*26*,*30*). Ensuring that future reviews also have a sufficient range of expertise will be important.

The number of experts participating in the annual review meeting also deserves careful consideration. Larger groups increase the likelihood of reproducibility and decrease the risk for certain biases (*57*). Smaller groups can simplify the consensus-building process (*15*,*58*). Group size can also impact group dynamics. Too large a group can make face-to-face consultations impractical, complicating efforts to review and discuss the results, correct eventual inconsistencies, reach consensus, and avoid misunderstanding (*8*). Although exploring ways that a greater number of experts might be involved with developing an initial long list of diseases to be considered might be useful, a more limited group will probably need to continue to analyze the short list in the years to come.

Additional efforts are also needed to address recall bias. Discussions during the 2017 review highlighted that the diseases that enjoyed the greatest levels of support for inclusion in the revised priority list had all caused recent major outbreaks. An annual “landscape review” (in which each disease on the long list of proposed diseases is independently reviewed, considering factors such as the current knowledge regarding prioritization criteria, risk for emergence, and availability of countermeasures, regardless of recent events) should contribute to avoiding a disproportionate emphasis based on recent events. In the short term, participants in the next annual review should be briefed on, and discuss the impact of, recall bias before undertaking the MCDA scoring exercise. In the longer term, options for weighting against recent public health emergencies might be explored, perhaps through the development of a calibration curve, which has been used to mitigate recall bias in other types of processes (*59*).

This methodology is expert-driven, and despite all efforts to minimize biases related to their efforts, biases still occur. To address this problem, WHO should 1) change the composition of the prioritization committees yearly and expand the geographic range of the experts involved and 2) review the methodology separately with different experts.

The similarity between the WHO list of prioritized diseases and those found in other studies suggests a degree of consistency with previous findings (*8*–*11*,*13*,*14*,*35*,*41*). The results of the sensitivity analysis demonstrate that the R&D Blueprint ranking is robust, corresponding with earlier observations that the analytic hierarchy process is not sensitive to minor changes in criteria weights (*55*). Even when major changes on the weight of criteria were applied, the final ranking remained largely stable. Throughout all the scenarios used in this sensitivity analysis, the same 3 groupings of diseases remained consistent. In some scenarios, the ranking of diseases within the group changed, but this observation is consistent with the findings of other prioritization exercises (*9*). Being able to produce a similar 3-tiered group ranking using another model, the SMART Vaccines prioritization tool, also suggests that the approach employed for the R&D Blueprint is producing valid results.

However, the impact of the MCMs criterion needs further consideration. The sensitivity analysis showed that the contribution of this criterion to the final ranking is limited despite its high weight. This observation is probably explained by the objectives of the R&D Blueprint itself, which focuses on diseases for which few or no MCMs exist, meaning that all the diseases considered score equally in this regard. Ensuring that sufficient attention is paid to this issue when selecting diseases for inclusion on the long-list will be useful as a screening process. Ensuring that distinct R&D gaps are a prerequisite for inclusion could result in this criterion being dropped from the MCDA.

In conclusion, the R&D Blueprint fills a considerable gap in public health preparedness by supporting R&D on highly infectious diseases for which few or no countermeasures exist. To translate this objective into effective action, WHO had to determine the diseases that most urgently required the commencement of work. For each of these priority diseases, WHO is developing roadmaps; target product profiles for vaccines, therapeutics, and diagnostics (*60*); and generic protocols for vaccine and therapeutic clinical trials. The R&D Blueprint is also enabling cross-cutting support activities, such as data and sample sharing norms, regulatory preparedness aspects, and overall research coordination (*61*). Aware of the shortcomings of past efforts to develop similar lists, WHO explored lessons learned and best practices for developing a new approach. The challenge was in balancing competing needs for a standardized, robust methodology that can be repeated on a regular basis, with a reliance on expert opinion. Because this methodology and its supporting tools will be subjected to a full review within 2 years, WHO hopes that the lessons learned through the R&D Blueprint’s repeated use, including those we have identified, will be used to improve it further.

Technical Appendix 1 Three components of the World Health Organization R&D Blueprint prioritization methodology.

Technical Appendix 2 Prioritization criteria considered in the development of the World Health Organization R&D Blueprint.

Technical Appendix 3 Diseases prioritization methodologies and their applications since 2015.

Technical Appendix 4 Multicriteria decision analysis used in the development of the World Health Organization R&D Blueprint.

Technical Appendix 5 Multicriteria scores calculation and detailed discordance estimation procedure used in the development of the World Health Organization R&D Blueprint.

Technical Appendix 6 Additional considerations of diseases not incorporated into the final list in the development of the World Health Organization R&D Blueprint.
