# Supplementary material for: World Health Organization Methodology to Prioritize Emerging Infectious Diseases in Need of Research and Development
Source: Emerg Infect Dis. 2018 Sep;24(9):e171427. doi: 10.3201/eid2409.171427 (PMC6106429; doi:10.3201/eid2409.171427)
Supplement: Technical Appendix 1 — Three components of the World Health Organization R&D Blueprint prioritization methodology. [file 17-1427-Techapp-s1.pdf]

Article DOI: https://doi.org/10.3201/eid2409.171427

World Health Organization Methodology to Prioritize Emerging Infectious Diseases in Need of Research and Development

Technical Appendix 1

The three components of the Blueprint prioritization methodology

1. The annual review:

• Convening a suitable expert group (Prioritization Committee, Table) covering: 1) microbiology of severe pathogens, including virology, bacteriology and mycology, 2) clinical management of severe infections, 3) Epidemiology, in particular during health emergencies, 4) Public health policy, including emergency response, 5) Animal health, including veterinarians and experts in zoonoses from both livestock and wildlife, 6) experts from the defense or security sectors familiar with biological weapons and 7) other experts, including anthropologists, bioethicists, and other relevant social sciences.

• Identifying a long list of diseases to be fed into the annual review process.

• Triaging the long list into a shorter list for more detailed analysis.

• Conducting that analysis through the Analytic Hierarchy Process (AHP)/Multi-criteria Decision Analysis (MCDA) method and Delphi process.

• Communicating the outcome of the review.

Technical Appendix 1 Table. Prioritization Committee

Members	Sex	WHO region*
Prioritization Committee		
Dr. Celia ALPUCHE	Female	PAHO
Prof. Lucille BLUMBERG	Female	AFRO
Dr. David BRETT-MAJOR	Male	PAHO
Dr. Miles CAROLL	Male	EURO
Dr. Inger DAMON	Female	EURO
Dr. Peter DASZAK	Male	PAHO
Dr. Xavier DE LAMBALLERIE	Male	PAHO
Dr. Mourya DEVENDRA	Male	SAERO
Prof. Christian DROSTEN	Male	EURO
Dr. Delia ENRIA	Female	PAHO
Prof. Sahr GEVAO	Male	AFRO
Prof. Stephan GUENTHER	Male	EURO
Prof. Peter HORBY	Male	EURO
Prof. Roger HEWSON	Male	EURO
Dr. Nadia KHELEF	Female	EURO
Prof. Gary KOBINGER	Male	PAHO
Dr. Linda LAMBERT	Female	PAHO
Dr. Dieudonne NKOGHE	Male	AFRO
Dr. George WARIMWE	Male	AFRO
Dr. Mark WOOLHOUSE	Male	EURO
Dr. Youngmee Jee	Female	WIPRO
Dr. Stefano MESSORI	Male	EURO
Dr. Cathy ROTH	Female	EURO
Dr. Heinz FELDMANN	Male	PAHO
Observers		
Dr. Hinta MEIJERINK	Female	Coalition of Epidemic Preparedness Innovation, EURO
Ms. Stacey KNOBLER	Female	U.S. National Institutes of Health, PAHO
Dr. Ben MCCORMICK	Male	U.S. National Institutes of Health, PAHO

*EURO: European region, PAHO: American region, SAERO: south-east Asian region, AFRO: African region, WIPRO: West Pacific region. Detailed country members: http://www.who.int/about/regions/en.

2. The methodology review

In accordance with best practice, separate processes were used to develop the methodology and run the annual review (*1*–*5*). According to Brookes et al. 2015, separating these processes improves transparency "*by clearly separating decision-makers subjective opinions regarding the value of criteria from measurements for individual pathogens, as well as reducing opportunity for cognitive bias that can arise when directly valuing pathogens*" (*5*). In addition to the annual exercise to update the list, the methodology itself will be reviewed every 2 years. This methodology review involves: convening a group of suitable experts; examining and revising the prioritization criteria and sub-criteria; and updating the weightings applied to the criteria.

3. Decision tree

The broader prioritization process also includes a decision tree for consideration of an unknown disease or a known disease presenting with unusual characteristics. The decision instrument is intended to guide users through: considering available information, determining

whether an emergency prioritization review is warranted, and whether this disease should be considered for the next annual review.

References

1. Cardoen S, Van Huffel X, Berkvens D, Quoilin S, Ducoffre G, Saegerman C, et al. Evidence-based semiquantitative methodology for prioritization of foodborne zoonoses. Foodborne Pathog Dis. 2009;6:1083–96. PubMed http://dx.doi.org/10.1089/fpd.2009.0291

2. Balabanova Y, Gilsdorf A, Buda S, Burger R, Eckmanns T, Gärtner B, et al. Communicable diseases prioritized for surveillance and epidemiological research: results of a standardized prioritization procedure in Germany, 2011. PLoS One. 2011;6:e25691. PubMed http://dx.doi.org/10.1371/journal.pone.0025691

3. Humblet M-F, Vandeputte S, Albert A, Gosset C, Kirschvink N, Haubruge E, et al. Multidisciplinary and evidence-based method for prioritizing diseases of food-producing animals and zoonoses. Emerg Infect Dis. 2012;18:e1. PubMed http://dx.doi.org/10.3201/eid1804.111151

4. European Centre for Disease Prevention and Control. Best practices in ranking emerging infectious disease threats: a literature review [cited 2015 Feb 1]. https://ecdc.europa.eu/sites/portal/files/media/en/publications/Publications/emerging-infectious-disease-threats-best-practices-ranking.pdf

5. Brookes VJ, Del Rio Vilas VJ, Ward MP. Disease prioritization: what is the state of the art? Epidemiol Infect. 2015;143:2911–22. PubMed http://dx.doi.org/10.1017/S0950268815000801
