# Supplementary material for: World Health Organization Methodology to Prioritize Emerging Infectious Diseases in Need of Research and Development
Source: Emerg Infect Dis. 2018 Sep;24(9):e171427. doi: 10.3201/eid2409.171427 (PMC6106429; doi:10.3201/eid2409.171427)
Supplement: Technical Appendix 2 — Prioritization criteria considered in the development of the World Health Organization R&D Blueprint. [file 17-1427-Techapp-s2.pdf]

Article DOI: https://doi.org/10.3201/eid2409.171427

World Health Organization Methodology to Prioritize Emerging Infectious Diseases in Need of Research and Development

Technical Appendix 2

Prioritization Criteria

All three components of the prioritization process make use of a common set of criteria and sub-criteria. The criteria represent top level factors which might impact the relevance of a disease to the R&D Blueprint, such as the human transmissibility of the disease, or the societal impact. The sub-criteria then explore different facets of each of these areas, for example considering different types of countermeasures or their suitability for use in different resource settings.

In advance of the 2015 consultation, WHO reviewed criteria and sub-criteria used in earlier disease prioritization exercises. The results were included in background materials for the consultation (*1*). Nine prioritization criteria and numerous sub-criteria were identified through moderated discussions. Following feedback from the R&D Blueprint's Scientific Advisory Group in May 2016, and the subsequent work of the methodology review meeting in November 2016, the original nine criteria were compressed into the current eight criteria below to insure completeness, non-redundancy, nonoverlapping and preference independence (*2*).

1. Human Transmission

Subcriteria

a) There is evidence of human to human transmission

b) There is widespread human to human transmission

c) There is more than one route of human to human transmission

d) The disease frequently involves infectivity before the onset of symptoms

e) The pathogen is able to remain infectious for a prolonged period in an infected individual when convalescent or apparently recovered

f) There is evidence of superspreading events

g) The disease is likely to be amplified in a healthcare setting

2. Medical Countermeasures

Subcriteria

a) Diagnostics which are effective and suitable for use in the field are not available

b) Diagnostics which are effective and suitable for use in a clinic or local healthcare setting are not available

c) Effective Diagnostics are available but are only suitable for use in specialized facilities

d) Effective vaccines (human or animal, as appropriate) and prophylactics do not exist

e) Effective vaccines (human or animal, as appropriate) and prophylactics which are suitable for use in resource limited settings do not exist

f) Effective drugs or therapies do not exist

g) Effective drugs or therapies which are appropriate for use in resource limited settings do not exist

h) The outbreak cannot be controlled by the application of common public health measures (such as contact tracing, Isolation of infected patients, social distancing, closure of public events, schooling, changes to cultural practices, e.g., burial rights, vector control, strict management of livestock movement)

3. Severity or Case-Fatality Rate

Subcriteria

a) The disease causes high mortality

b) The disease frequently causes high morbidity, including severe complications or sequelae

4. The Human–Animal Interface

Subcriteria

a) The involvement of animals in transmitting (including arthropods) the disease to people is well characterized

b) There are transmission routes from animals (including arthropods) to humans likely to result in high levels of human infections

c) The pathogen is capable of infecting multiple animal species

d) The animal species transmitting the disease are widely distributed

e) The animal species transmitting the disease is abundant

f) Arthropod(s) are responsible for transmitting the disease

g) Arthropod(s) responsible for transmitting the disease are widely distributed

5. Other Factors

Subcriteria:

a) The geographic range of the pathogen has changed

b) The pathogen shares relevant epidemiologic and/or genotypic characteristics with agents which have caused important epidemics

c) The natural disease does not result in robust protective immunity

d) The disease carries a high risk of occupational exposure for those involved in a response (including for culling, vets, burial details, lab workers, first responders, healthcare workers)

e) The pathogen is an agent likely to be used to cause deliberate outbreaks

6. The Public Health Context of the Affected Area

Subcriteria

a) The disease requires targeted surveillance (i.e., not likely to be detected by routine surveillance but which might be detected by active or sentinel surveillance)

b) Disease control requires specialist interventions (such as highly skilled personnel; equipment, such as isolation units, respirators, PPE, etc.; and infection control measures)

7. Potential Societal Impacts

Subcriteria

a) The disease has a disproportionate impact on special populations (such as pregnant women, children, immunocompromised, etc.)

b) The disease can cause major social disruption

c) The disease can cause major fear

d) The disease can result in major economic impact

e) The disease can result in a major disruption to healthcare delivery

8. The Evolutionary Potential of the Pathogen

Subcriteria

a) There is evidence of rapid pathogen evolution

b) There is a trend toward increasing severity of the disease

c) There is a trend toward the increasing transmissibilty of the pathogen

From the outset, there was an understanding that these different criteria may not have an equal impact on relevance to the R&D Blueprint and therefore whether a disease needs to be prioritized. As an initial step, the 2015 consultation experts ranked the criteria they identified but recommended a semiquantitative approach be developed (*1*). During 2016 WHO developed the current process which uses the Analytic Hierarchy Process (AHP) to undertake a pair-wise review of criteria. Experts participating in the November 2016 Methodology Review committee were surveyed as to the relative importance of the prioritization criteria (*3*). The overall 2016 criteria weights are in Technical Appendix 2 Figure.

References

1. World Health Organization. WHO publishes list of top emerging diseases likely to cause major epidemics [cited 2015 Dec 10]. http://www.who.int/medicines/ebola-treatment/WHO-list-of-top-emerging-diseases/en/

2. Marsh K, IJzerman M, Thokala P, Baltussen R, Boysen M, Kaló Z, et al.; ISPOR Task Force. Multiple criteria decision analysis for health care decision making—emerging good practices: report 2 of the ISPOR MCDA Emerging Good Practices Task Force. Value Health. 2016;19:125–37. PubMed http://dx.doi.org/10.1016/j.jval.2015.12.016

3. World Health Organization. R&D Blueprint: progress in prioritizing diseases with epidemic potential [cited 2016 Nov 18]. http://www.who.int/blueprint/what/research-development/prioritizing_diseases_progress/en/

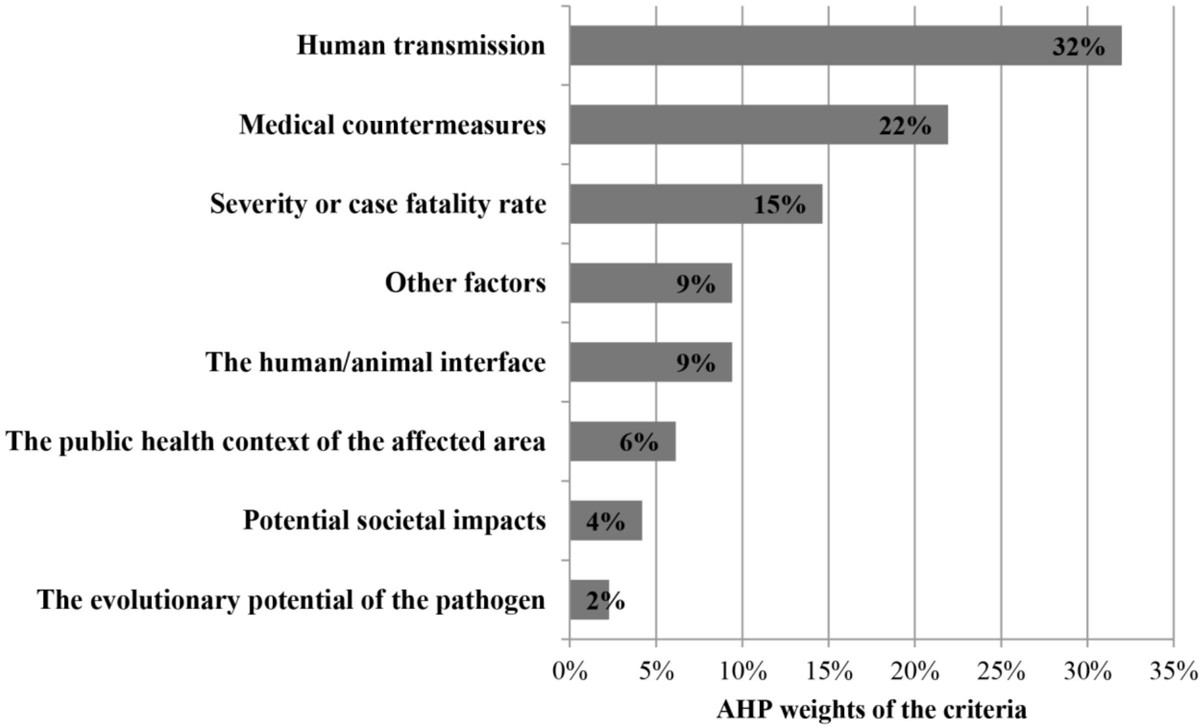

Technical Appendix 2 Figure. Relative importance of the criteria as indicated by their weights.
