# Supplementary material for: World Health Organization Methodology to Prioritize Emerging Infectious Diseases in Need of Research and Development
Source: Emerg Infect Dis. 2018 Sep;24(9):e171427. doi: 10.3201/eid2409.171427 (PMC6106429; doi:10.3201/eid2409.171427)
Supplement: Technical Appendix 3 — Diseases prioritization methodologies and their applications since 2015. [file 17-1427-Techapp-s3.pdf]

Article DOI: https://doi.org/10.3201/eid2409.171427

World Health Organization Methodology to Prioritize Emerging Infectious Diseases in Need of Research and Development

Technical Appendix 3

Disease Prioritization Methodologies and Their Application Since 2015

Technical Appendix 3 Table. Recent disease prioritization methodologies and their application.

Study	Country	Purpose of the prioritization	Tools used in the methodology
Stebler et al. 2015 (*1*)	Switzerland	Zoonotic diseases prioritization in Switzerland for surveillance and control	Semiquantitative Delphi process
Garner et al. 2015 (*2*)	Canada	Methodological study for antimicrobial resistant disease risk prioritization and its application in Canada	Multi-Criteria Decision Analysis (MCDA)–PROMETHEE
Dahl et al. 2015 (*3*)	Sweden	Pathogens Prioritization according to their public health prevalence for resource allocation in Sweden	Delphi process with weighting
Ciliberti et al. 2015 (*4*)	EU	Prioritization of wildlife pathogens for surveillance programs in the EU	MCDA
Kadohira et al. 2015 (*5*)	Japan	Methodology for zoonosis prioritization and application for risk assessment in Japan	Risk profiling and AHP
M Bouwknegt et al. 2015 (*6*)	EU	“Risk ranking study to identify emerging diseases that could pose threats to the health and security of the EU”	MCDA
Siembieda et al. 2015 (*7*)	Vietnam	Zoonotic diseases prioritization in Vietnam for resource allocation	Questionnaire
Hongoh et al 2016 (*8*)	Canada (Quebec and Burkina Faso)	Infectious disease prioritization related to climate in Quebec and Burkina Faso for resource allocation	MCDA–PROMETHEE
Lapid et al. 2016 (*9*)	Israel	Prioritization of wildlife pathogens for surveillance in Israel	Rapid Risk Analysis
Stebler et al. 2016 (*10*)	Switzerland	Compare the zoonotic disease prioritization by students and public health professionals in Switzerland	Co-joint analysis questionnaires
McFadden et al. 2016 (*11*)	Mongolia	Zoonotic diseases prioritization for resource allocation	Multi-criteria ranking model
Brioudes al. 2016 (*12*)	Pacific Island countries and territories (PICTs)	Identification of animal pathogens for animal health resources allocation	Delphi process
Munyua et al. 2016 (*13*)	Kenya	Zoonotic diseases prioritization in Kenya for prevention and control strategies in Kenya	Analytic Hierarchy process (AHP)–Decision tree
Pieracci et al. 2016 (*14*)	Ethiopia	Zoonotic diseases prioritization in Ethiopia for prediction, prevention and response	AHP–Decision tree

References

1. Stebler N, Schuepbach-Regula G, Braam P, Falzon LC. Use of a modified Delphi panel to identify and weight criteria for prioritization of zoonotic diseases in Switzerland. Prev Vet Med. 2015;121:165–9. PubMed http://dx.doi.org/10.1016/j.prevetmed.2015.05.006

2. Garner MJ, Carson C, Lingohr EJ, Fazil A, Edge VL, Trumble Waddell J. An assessment of antimicrobial resistant disease threats in Canada. PLoS One. 2015;10:e0125155. PubMed http://dx.doi.org/10.1371/journal.pone.0125155

3. Dahl V, Tegnell A, Wallensten A. Communicable diseases prioritized according to their public health relevance, Sweden, 2013. PLoS One. 2015;10:e0136353. PubMed http://dx.doi.org/10.1371/journal.pone.0136353

4. Ciliberti A, Gavier-Widén D, Yon L, Hutchings MR, Artois M. Prioritisation of wildlife pathogens to be targeted in European surveillance programmes: Expert-based risk analysis focus on ruminants. Prev Vet Med. 2015;118:271–84. PubMed http://dx.doi.org/10.1016/j.prevetmed.2014.11.021

5. Kadohira M, Hill G, Yoshizaki R, Ota S, Yoshikawa Y. Stakeholder prioritization of zoonoses in Japan with analytic hierarchy process method. Epidemiol Infect. 2015;143:1477–85. PubMed http://dx.doi.org/10.1017/S0950268814002246

6. Bouwknegt M, Havelaar A, Neslo R, de Roda Husman AM, Hogerwerf L, van Steenbergen J, et al. Ranking infectious disease risks to support preparedness prioritization in the European Union: Jonathan Suk. Eur J Pub Health. 2015;25(Suppl 3):ckv167.036-ckv167.036.

7. Trang T, Siembieda J, Huong NT, Hung P, Ky VD, Bandyopahyay S, et al. Prioritization of zoonotic diseases of public health significance in Vietnam. J Infect Dev Ctries. 2015;9:1315–22. PubMed http://dx.doi.org/10.3855/jidc.6582

8. Hongoh V, Michel P, Gosselin P, Samoura K, Ravel A, Campagna C, et al. Multi-stakeholder decision aid for improved prioritization of the public health impact of climate sensitive infectious diseases. Int J Environ Res Public Health. 2016;13:419. PubMed http://dx.doi.org/10.3390/ijerph13040419

9. Lapid R, King R, Yakobson B, Shalom U, Moran-Gilad J. Wildlife pathogen surveillance in Israel to inform human and animal infectious disease control: a prioritization exercise. Isr J Vet Med. 2016;71:33–41.

10. Stebler N, Schuepbach-Regula G, Braam P, Falzon LC. Weighting of criteria for disease prioritization using conjoint analysis and based on health professional and student opinion. PLoS One. 2016;11:e0151394. PubMed http://dx.doi.org/10.1371/journal.pone.0151394

11. McFadden AM, Muellner P, Baljinnyam Z, Vink D, Wilson N. Use of multicriteria risk ranking of zoonotic diseases in a developing country: case study of Mongolia. Zoonoses Public Health. 2016;63:138–51. PubMed http://dx.doi.org/10.1111/zph.12214

12. Brioudes A, Warner J, Hedlefs R, Gummow B. Diseases of livestock in the Pacific Islands region: setting priorities for food animal biosecurity. Acta Trop. 2015;143:66–76. PubMed http://dx.doi.org/10.1016/j.actatropica.2014.12.012

13. Munyua P, Bitek A, Osoro E, Pieracci EG, Muema J, Mwatondo A, et al. Prioritization of zoonotic diseases in Kenya, 2015. PLoS One. 2016;11:e0161576. PubMed http://dx.doi.org/10.1371/journal.pone.0161576

14. Pieracci EG, Hall AJ, Gharpure R, Haile A, Walelign E, Deressa A, et al. Prioritizing zoonotic diseases in Ethiopia using a one health approach. One Health. 2016;2:131–5. PubMed http://dx.doi.org/10.1016/j.onehlt.2016.09.001
