# Supplementary material for: World Health Organization Methodology to Prioritize Emerging Infectious Diseases in Need of Research and Development
Source: Emerg Infect Dis. 2018 Sep;24(9):e171427. doi: 10.3201/eid2409.171427 (PMC6106429; doi:10.3201/eid2409.171427)
Supplement: Technical Appendix 4 — Multicriteria decision analysis used in the development of the World Health Organization R&D Blueprint. [file 17-1427-Techapp-s4.pdf]

Article DOI: https://doi.org/10.3201/eid2409.171427

World Health Organization Methodology to Prioritize Emerging Infectious Diseases in Need of Research and Development

Technical Appendix 4

Multicriteria Decision Analysis

MCDA techniques can be compensatory or non-compensatory (*1*). Compensatory MCDA allow trade-offs between criteria whereas non-compensatory do not. According to Baltussen and Niessen 2006, MCDA compensatory methods are more suitable for use for public health purpose (*2*). Several MCDA compensatory techniques have been used for the prioritization of infectious diseases (*3–11*). One such technique is the Analytic Hierarchy Process (AHP) developed by Thomas Saaty (*12*).

AHP uses pair-wise comparisons based on expert judgement that directly incorporates expert knowledge (*13*). Saito et al. 2015 highlighted the ability of the AHP to enable an expert group "*to make trade-off and establish priorities among qualitative and quantitative inputs*". This is particularly useful in animal and human health where many characteristics remain unclear or unknown (*13*).

Five past disease prioritization studies used AHP for criteria weighting but used different approaches for disease scoring (*8–11*,*14*). Zoonoses prioritization in Japan made use of a rating mode with absolute measures (*11*). A classical AHP scoring by pair-wise comparison was used for prioritization of animal infectious diseases in Chile (*9*). A decision tool to score diseases through a set of qualitative questions in the absence of expert opinion was developed by the CDC (*8*), and used recently in Kenya (*10*) and Ethiopia (*14*).

None of the past implementations of AHP were a good fit for the specific needs of the R&D Blueprint disease scoring. As a result, the WHO methodology includes a tailored

implementation of the AHP, using pair-wise comparisons for weighting criteria, but makes use of a different disease scoring process.

References

1. Jeffreys I. The use of compensatory and non-compensatory multi-criteria analysis for small-scale forestry. Small-scale For. 2004;3:99–117.

2. Baltussen R, Niessen L. Priority setting of health interventions: the need for multi-criteria decision analysis. Cost Eff Resour Alloc. 2006;4:14. PubMed http://dx.doi.org/10.1186/1478-7547-4-14

3. Cardoen S, Van Huffel X, Berkvens D, Quoilin S, Ducoffre G, Saegerman C, et al. Evidence-based semiquantitative methodology for prioritization of foodborne zoonoses. Foodborne Pathog Dis. 2009;6:1083–96. PubMed http://dx.doi.org/10.1089/fpd.2009.0291

4. Balabanova Y, Gilsdorf A, Buda S, Burger R, Eckmanns T, Gärtner B, et al. Communicable diseases prioritized for surveillance and epidemiological research: results of a standardized prioritization procedure in Germany, 2011. PLoS One. 2011;6:e25691. PubMed http://dx.doi.org/10.1371/journal.pone.0025691

5. Gilsdorf A, Krause G. Prioritisation of infectious diseases in public health: feedback on the prioritisation methodology, 15 July 2008 to 15 January 2009. Euro Surveill. 2011;16:19861. PubMed

6. Humblet MF, Vandeputte S, Albert A, Gosset C, Kirschvink N, Haubruge E, et al. Multidisciplinary and evidence-based method for prioritizing diseases of food-producing animals and zoonoses. Emerg Infect Dis. 2012;18. PubMed http://dx.doi.org/10.3201/eid1804.111151

7. Krause G; Working Group on Prioritization at Robert Koch Institute. How can infectious diseases be prioritized in public health? A standardized prioritization scheme for discussion. EMBO Rep. 2008;9(Suppl 1):S22–7. PubMed http://dx.doi.org/10.1038/embor.2008.76

8. Rist CL, Arriola CS, Rubin C. Prioritizing zoonoses: a proposed one health tool for collaborative decision-making. PLoS One. 2014;9:e109986. PubMed http://dx.doi.org/10.1371/journal.pone.0109986

9. Maino M, Pérez P, Oviedo P, Sotomayor G, Abalos P. The analytic hierarchy process in decisionmaking for caprine health programmes. Rev Sci Tech. 2012;31:889–97. PubMed http://dx.doi.org/10.20506/rst.31.3.2162

10. Munyua P, Bitek A, Osoro E, Pieracci EG, Muema J, Mwatondo A, et al. Prioritization of zoonotic diseases in Kenya, 2015. PLoS One. 2016;11:e0161576. PubMed http://dx.doi.org/10.1371/journal.pone.0161576

11. Kadohira M, Hill G, Yoshizaki R, Ota S, Yoshikawa Y. Stakeholder prioritization of zoonoses in Japan with analytic hierarchy process method. Epidemiol Infect. 2015;143:1477–85. PubMed http://dx.doi.org/10.1017/S0950268814002246

12. Saaty TL. The analytic hierarchy process: planning, priority setting, resource allocation. New York: McGraw-Hill International Book Company; 1980.

13. Saito EK, Shea S, Jones A, Ramos G, Pitesky M. A cooperative approach to animal disease response activities: Analytical hierarchy process (AHP) and vvIBD in California poultry. Prev Vet Med. 2015;121:123–31. PubMed http://dx.doi.org/10.1016/j.prevetmed.2015.06.001

14. Pieracci EG, Hall AJ, Gharpure R, Haile A, Walelign E, Deressa A, et al. Prioritizing zoonotic diseases in Ethiopia using a one health approach. One Health. 2016;2:131–5. PubMed http://dx.doi.org/10.1016/j.onehlt.2016.09.001
