# Supplementary material for: World Health Organization Methodology to Prioritize Emerging Infectious Diseases in Need of Research and Development
Source: Emerg Infect Dis. 2018 Sep;24(9):e171427. doi: 10.3201/eid2409.171427 (PMC6106429; doi:10.3201/eid2409.171427)
Supplement: Technical Appendix 5 — Multicriteria scores calculation and detailed discordance estimation procedure used in the development of the World Health Organization R&D Blueprint. [file 17-1427-Techapp-s5.pdf]

Article DOI: https://doi.org/10.3201/eid2409.171427

World Health Organization Methodology to Prioritize Emerging Infectious Diseases in Need of Research and Development

Technical Appendix 5

Multicriteria Scores Calculation and Detailed Discordance Estimation Procedure

1. Multicriteria Scores Calculation

Subcriteria Weights

The criteria weights were calculated following the standard AHP procedure. The subcriteria were considered at equal importance, hence the weight of subcriteria *f* was equal to the weight of the corresponding criteria divided by its number of subcriteria. These weights were gathered in the weighting vector $\boldsymbol{W_{sub}}$.

Diseases Scores

The disease scores were calculated by using the normalization procedure of the AHP as explained below.

Let D_{ef} be the vector of expert *e*'s answers for sub-criterion *f*, $\boldsymbol{D_{ef}} = \begin{pmatrix} d_{ef1} \\ \vdots \\ d_{efp} \end{pmatrix}$ where *p* was the number of diseases.

Let $\boldsymbol{A_{ef}}$ be the comparison matrix of expert *e* for the sub-criterion *f* ($f = 1,2,\dots s,$ where *s* is the total number of subcriteria). The matrix $\boldsymbol{A_{ef}}$ was built by using the answers in $\boldsymbol{D_{ef}}$ as explained in equation 1.

$$\boldsymbol{A_{ef}} = \begin{pmatrix} \boldsymbol{a_{ef\,11}} & \cdots & \boldsymbol{a_{ef1p}} \\ \vdots & \ddots & \vdots \\ \boldsymbol{a_{efp1}} & \cdots & \boldsymbol{a_{efpp}} \end{pmatrix} = (\boldsymbol{a_{efij}}) = \begin{cases} \boldsymbol{a_{efij} = 1 \; if \; i = j} \\ \boldsymbol{a_{efij} = d_{efi} - d_{efj} + 1 \; if \; d_{efi} - d_{efj} \geq 0} \\ \boldsymbol{a_{efij} = \frac{1}{1-(d_{efi}-d_{efj})} \; if \; d_{efi} - d_{efj} \leq 0} \\ \boldsymbol{a_{efij} = NA \; if \; d_{efi} = 0 \; or \; d_{efj} = 0} \end{cases} \quad \text{(Equation 1)}$$

Once these comparison matrices were built for each expert, they were averaged to the comparison matrices A_f. According to Saaty, the geometric mean should be used when aggregating people's opinions (*1*): "Two important issues in group decision making are: how to aggregate individual judgements in a group into a single representative judgement for the entire group and how to construct a group choice from individual choices. The reciprocal property plays an important role in combining the judgements of several individuals to obtain a single judgement for the group. Judgements must be combined so that the reciprocal of the synthesized judgements is equal to the syntheses of the reciprocals of these judgements. It has been proved that the geometric mean, not the frequently used arithmetic mean, is the only way to do that."

For this methodology, the arithmetic average was also used to compare the results and to estimate the confidence on the final ranking. For this purpose, the data were processed in a different way. First, when the expert answers "I do not know" to any of the subcriteria statements d_{efi} was set equal to *NA* then the data were arithmetically averaged to the vector d_f. If d_{fi} was equal to *NA*, it was set to *0*. The comparison matrices were then built by using equation 1.

After the averaging step, if some elements of matrix $\boldsymbol{A_f}$, a_{fij}, remained equal to *NA*, this meant that for the disease *i* or *j* the information was not known among the Prioritization Committee. Accordingly, we considered these diseases of equal importance for the sub-criterion $f\ (a_{fij} = 1)$. In future prioritization exercises, the method of Bozóki et al. (2010) (*2*) will be used to solve this issue.

The weighting vectors $\boldsymbol{W_f}$ of the diseases for the sub-criterion *f* were calculated by following the steps described in equations 2 and 3. For the sake of clarity, the weighting vectors of the diseases were named scoring vectors.

The normalized comparison matrices of $\boldsymbol{A_f}$, $\boldsymbol{B_f}$, were computed by equation 2.

$$\boldsymbol{B_f} = (b_{fij}) = \left(\frac{a_{fij}}{\sum_i a_{fij}}\right) \text{ (Equation 2)}$$

The scoring vectors (an approximation of the principal eigenvector of matrix $\boldsymbol{A_f}$), $\boldsymbol{W_f} = \begin{pmatrix} w_{f1} \\ \vdots \\ w_{fn} \end{pmatrix}$,

of the diseases for the sub-criterion *f* were calculated by using equation 3.

$$\boldsymbol{w_{fi}} = \frac{\sum_j \boldsymbol{b_{fij}}}{\boldsymbol{n}} \text{ (Equation 3)}$$

Consistency Analysis

Once the scoring vectors computed, the consistency of this procedure was analyzed by calculating the consistency vectors, $\boldsymbol{C_v}$ as shown in equations 4 and 5.

$\boldsymbol{C} = \mathbf{A} \times \boldsymbol{W_f} = (\boldsymbol{c_f})$ (Equation 4)

$\mathbf{Cv} = (\boldsymbol{c_{v_f}}) = (\frac{\boldsymbol{c_f}}{\boldsymbol{w_f}})$ (Equation 5)

$\Lambda_{\mathbf{max}} = \frac{\sum_f^n \boldsymbol{c_{v_f}}}{\boldsymbol{n}}$ (Equation 6)

Where Λ_{max} was the maximum averaged eigenvalue. As $\boldsymbol{W_f}$ was an approximation of the eigenvector of matrix $\boldsymbol{A_f}$, $\boldsymbol{A_f} \times \boldsymbol{W_f} = \lambda_{max} \times \boldsymbol{W_f}$ where λ_{max} was the eigenvalue of the matrix $\boldsymbol{A_f}$. If the comparison was completely consistent then $\Lambda_{max} = \lambda max = n$. Hence, the difference between Λ_{max} and λmax represented the lack of consistency. To measure inconsistency, the coherence index was computed by using equation 7.

$\boldsymbol{CI} = \frac{\Lambda_{\mathbf{max}} - \boldsymbol{n}}{\boldsymbol{n} - \mathbf{1}}$ (Equation 7)

The higher the *CI*, the more incoherent the comparison and the weighting were. Thomas L. Saaty introduced by experimentation a coherence ratio *CR*, equation 8, to give a reference for the coherence analysis. If *CR* was higher than 10% then the comparison and the weighting were not consistent.

$\boldsymbol{CR} = \frac{\boldsymbol{CI}}{\boldsymbol{RI}}$ (Equation 8)

Where *RI* is the random inconsistency index of a matrix of order n. This analysis can be explained as the level of random comparisons in matrix $\boldsymbol{A_f}$. If *CR* is low, then matrix $\boldsymbol{A_f}$ was filled logically through a scale and rational analysis. If *CR* was high, then matrix $\boldsymbol{A_f}$ was filled randomly.

Multicriteria Scores

The final step of this process was to compute the multicriteria scores to rank the diseases. These multicriteria scores were computed by gathering the scoring vectors $\boldsymbol{W_f}$ in a matrix, $\boldsymbol{T}$, and by multiplying it by the weighting vector of the subcriteria $\boldsymbol{W_{sub}}$ as explained in equations 9 and 10.

$$\boldsymbol{T} = \begin{pmatrix} \boldsymbol{t_{11}} & \cdots & \boldsymbol{t_{1n}} \\ \vdots & \ddots & \vdots \\ \boldsymbol{t_{p,1}} & \cdots & \boldsymbol{t_{pn}} \end{pmatrix} \textbf{\textit{ where }} \boldsymbol{W_f} = \begin{pmatrix} \boldsymbol{t_{1f}} \\ \vdots \\ \boldsymbol{t_{pf}} \end{pmatrix} \text{(Equation 9)}$$

$$\boldsymbol{M} = \boldsymbol{T} \times \boldsymbol{W_{sub}} \text{ (Equation 10)}$$

Where ***M*** was the multicriteria score vector. This vector ranked the diseases according to their level of priority given the eight prioritization criteria. The disease with the highest score was the one with the highest priority. The disease with the lowest score was the one with lowest priority.

2. Detailed Discordance Estimation Procedure

Let $\boldsymbol{\Sigma D_f}$ be the vector of standard deviation of the vector $\boldsymbol{D_f}$, $\boldsymbol{\Sigma D_f} = \begin{pmatrix} \sigma_{d_{f1}} \\ \vdots \\ \sigma_{d_{fp}} \end{pmatrix}$

Let $\boldsymbol{\Sigma A_f}$ be the matrix of standard deviation of the matrix $\boldsymbol{A_f}$.

$$\Sigma \mathrm{A}_f = \begin{pmatrix} \boldsymbol{\sigma_{a_{f11}}} & \cdots & \boldsymbol{\sigma_{a_{f1p}}} \\ \vdots & \ddots & \vdots \\ \boldsymbol{\sigma_{a_{fp1}}} & \cdots & \boldsymbol{\sigma_{a_{fpp}}} \end{pmatrix} = \left(\boldsymbol{\sigma_{a_{fij}}}\right) = \begin{cases} \boldsymbol{if\ i = j:\ \sigma_{a_{fij}} = 0} \\ \boldsymbol{if\ d_{fi} = 0\ or\ d_{fj} = 0:\ \sigma_{a_{fij}} = 0} \\ \textbf{Else}: \boldsymbol{\sigma_{a_{fij}} = \sqrt{\sigma^2_{d_{fi}} + \sigma^2_{d_{fi}}}} \end{cases} \text{(Equation 11)}$$

Thus the discordance on the normalized matrix $\boldsymbol{B_f}$, $\boldsymbol{\Sigma B_f}$, was given by equation 12.

$$\begin{cases} \boldsymbol{\Sigma \mathrm{B}_f} = \begin{pmatrix} \boldsymbol{\sigma_{b_{f11}}} & \cdots & \boldsymbol{\sigma_{b_{f1p}}} \\ \vdots & \ddots & \vdots \\ \boldsymbol{\sigma_{b_{fp1}}} & \cdots & \boldsymbol{\sigma_{b_{fpp}}} \end{pmatrix} = \left(\boldsymbol{\sigma_{b_{fij}}}\right) \\ \boldsymbol{\sigma_{b_{fij}} = b_{fij} \sqrt{\frac{\sigma_{a_{fij}}^2}{a_{fij}^2} + \frac{\sum_i \sigma_{a_{fij}}^2 + 2 \times \sum_{l<k} \sigma_{(a_{flj})(a_{fkj})}}{\left(\sum_i a_{fij}\right)^2}}} \end{cases} \text{(Equation 12)}$$

Where $\sigma_{(a_{flj})(a_{fkj})}$ measured the dependence of the variables a_{flj}, a_{fkj}.

The discordance on the weighting vectors, $\boldsymbol{W_f}$, of the diseases for the criterion f was given by equation 13.

$$\begin{cases} \boldsymbol{\Sigma W_f} = \begin{pmatrix} \boldsymbol{\sigma_{t_{1f}}} \\ \vdots \\ \boldsymbol{\sigma_{t_{pf}}} \end{pmatrix} \\ \boldsymbol{\sigma_{t_i} = \sqrt{\frac{\sum_i^p \sigma_{\mathrm{b}_{fij}}^2 + 2 \times \sum_{l<k} \sigma_{(\mathrm{b}_{fil})(\mathrm{b}_{fik})}}{p^2}}} \end{cases} \text{(Equation 13)}$$

Where $\sigma_{(\mathrm{b_{fim}})(\mathrm{b_{fik}})}$ measured the dependence of the variables $\mathrm{b_{fim}}, \mathrm{b_{fik}}$.

The discordance on the final prioritization scores were computed by using the error propagation technique from matrix **T** to matrix **M** through equation 10. The discordance on the vector **M**, **ΣM**, was given by equations 14 and 15.

$$\boldsymbol{\Sigma M} = \begin{pmatrix} \boldsymbol{\sigma_{m_1}} \\ \vdots \\ \boldsymbol{\sigma_{m_p}} \end{pmatrix} \text{(Equation 14)}$$

Where

$$\boldsymbol{\sigma_{m_i}} = \sqrt{\sum_f^n \mathbf{w}_f^2 \times \boldsymbol{\sigma}_{t_{if}}^2 + 2 \times \sum_{l<k} \mathbf{w}_l \mathbf{w}_k \boldsymbol{\sigma}_{(t_{ih})(t_{ik})}} \text{(Equation 15)}$$

References

1. Saaty TL. Decision making with the analytic hierarchy process. Int J Serv Sci. 2008;1:83–98. http://dx.doi.org/10.1504/IJSSCI.2008.017590

2. Bozóki S, Fülöp J, Rónyai L. BozóKi S. Fülöp J, RóNyai L. On optimal completion of incomplete pairwise comparison matrices. Math Comput Model. 2010;52:318–33. http://dx.doi.org/10.1016/j.mcm.2010.02.047
