# Supplementary material for: World Health Organization Methodology to Prioritize Emerging Infectious Diseases in Need of Research and Development
Source: Emerg Infect Dis. 2018 Sep;24(9):e171427. doi: 10.3201/eid2409.171427 (PMC6106429; doi:10.3201/eid2409.171427)
Supplement: Technical Appendix 6 — Additional considerations of diseases not incorporated into the final list in the development of the World Health Organization R&D Blueprint. [file 17-1427-Techapp-s6.pdf]

Article DOI: https://doi.org/10.3201/eid2409.171427

World Health Organization Methodology to Prioritize Emerging Infectious Diseases in Need of Research and Development

Technical Appendix 6

Additional Consideration of Diseases Not Incorporated into the Final List in the Development of the World Health Organization R&D Blueprint

The meeting noted that several diseases discussed during the review, such as dengue, yellow fever, HIV/AIDS, tuberculosis, malaria, avian influenza causing severe human disease, antimicrobial resistance, and smallpox/monkeypox, continue to pose major public health problems and further research and development is needed. In this regard, participants recognized the existence of major disease control initiatives, extensive R&D pipelines, existing funding streams, or established regulatory pathways for improved interventions for these diseases, so they were ultimately excluded from the R&D Blueprint priority list.

Several additional pathogens were discussed and considered for inclusion in a priority list, such as: emerging flaviviruses with potential for hemorrhagic fever (such as Kyasanur Forest Disease) or those with potential for encephalitis (such as Usutu); emerging Bunyaviruses (such as Oropouche); emerging Alphaviruses (such as Chikungunya and Mayaro virus); rickettsia; plague; hantaviral diseases; and Chandipura virus disease. It was noted that a potential threat need not be a virus and could be any type of pathogen. For several of these diseases more research is needed before even an assessment for prioritized countermeasure development can be undertaken. Necessary research might include basic/fundamental and characterization research, as well as epidemiologic or entomological studies, or further elucidation of transmission routes. In some cases existing tools may need to be improved.

Certain types of cross-cutting research and development should be encouraged for the management of prioritized diseases and other potential public health threats, including a novel or deliberate threat. Participants highlighted the importance of validated diagnostic tests (including

differential diagnosis), tools for identifying the cause of syndromes, as well as diverse countermeasures that work across different pathogens or diseases, including vector control.

The value of a One Health approach was also stressed – both in terms of parallel prioritization processes to support research and development against animal diseases and joint efforts for pathogens in common. The possible utility of animal vaccines for preventing public health emergencies was also noted.

Although anti-microbial resistance is addressed through specific international initiatives the possibility was not excluded that in the future, a resistant pathogen might emerge and appropriately be prioritized, as a specific threat.
